# A Framework of Industrialized Building Assessment in China Based on the Structural Equation Model

**DOI:** 10.3390/ijerph15081687

**Published:** 2018-08-08

**Authors:** Lei Jiang, Zhongfu Li, Long Li, Tiankun Li, Yunli Gao

**Affiliations:** 1 Department of Construction Management, Dalian University of Technology, Dalian 116024, China; jianglei@dlnu.edu.cn (L.J.); lizhongfu@dlut.edu.cn (Z.L.); 2 School of Civil Engineering, Dalian Minzu University, Dalian 116650, China; yunligao@163.com; 3 Operation Management Department, Tahoe Real Estate Group, Beijing 100020, China; leetka@163.com

**Keywords:** industrialized building (IB), industrialized building assessment (IBA), exploratory factor analysis (EFA), confirmatory factor analysis (CFA), structural equation model (SEM), China

## Abstract

Compared with the conventional building, the industrialized building (IB) promotes the sustainable development of the construction industry, which will become a growth trend in the future. Nevertheless, the progress of industrialized building is intimately affected through the scientific evaluating mechanism, which still requires more research. Thus, this study establishes a conceptual framework of industrialized building assessment (IBA), which is validated through exploratory factor analysis (EFA) and confirmatory factor analysis (CFA). The impact between efficiency and the other five dimensions are studied by the structural equations model (SEM). The findings indicated that the conceptual framework is valid, and the efficiency has a positive impact on economic factors, livability, safety, environmental factors, and social benefits. Consequently, the improvement of efficiency has turned out to be the primary issue for improving the growth of the industrialized building. This research explores the basic framework of industrialized building assessment and provides a basis to establish a comprehensive and precise industrial building evaluation mechanism in the near future.

## 1. Introduction

The degree of industrialization in various sectors has been promoted rapidly; in contrast, the industrialization of the construction industry has manifested slower growth. Conventional construction is inefficient with extensive energy consumption [1,2,3], causing serious damage to the environment [4,5]. In the implementation process of industrialized building (IB), components could be produced in factories [6,7], then transported to assemble on site mechanically [8]. In addition, construction industrialization offers greater advantages as compared with the conventional construction methods [9], which could enhance labor productivity [10] and quality [11], together with lowering the labor force [11], saving energy, and safeguarding the environment [12].

The concept of IB stems from manufacturing based on large-scale production, integrating the production mechanism of prefabrication, mechanized production, automatic production, robot production, and replication [13]. IB deals with the use of not only the standardized design [14], but also industrialized production methods [15], prefabrication in factories [16], state-of-the-art mechanical equipment for on-site assembly [17], and scientific organization methods for the management and construction of buildings [18]. In comparison with the conventional building, IB is capable of not just substantially improving the efficiency of production [19,20], shortening the construction period, and improving the quality [21,22], but also it lowers environmental pollution, as well as the wasting of resources [23], in addition to improving the environment of the construction site [24] and increasing the construction safety [25].

With the development of IB, many researchers have emphasized the importance of industrialized building assessment (IBA). The preliminary research primarily dealt with single-aspect assessment, for instance, economic aspects [26,27], environmental aspects [28], and social aspects [29,30]. Subsequent to that, the IBA research has become more extensive, covering multiple aspects. For instance, Pons and Wadel adopted a life cycle assessment for the determination of the level of quality enhancement and environmental pollution minimization of IB [31]. Aye et al. performed an evaluation of the potential environmental and social benefits resulting from the reuse of materials, minimization of landfill use, and resource demand [32]. Nevertheless, there exists little research addressing IBA, as well as the impact of different aspects on the assessment. A rational evaluation system is essential for industrialization in the preliminary stage. Thus, to establish a scientific IBA, a conceptual framework is required for promoting the growth of IB in China.

Since 2015, China has allotted various policies to support IB and has enhanced the corresponding technical standard system, which has encouraged the rapid development of newly started areas of IB [26]. Nevertheless, the proportion of IB in new construction is approximately 5%, which is far behind that in the industrialized countries [33]. Moreover, the current technology and standard system of IB are not flawless enough, which has extremely restricted building industrialization. Mostly, the IBA of China is concentrated on the computation of an assembly rate, which cannot efficiently assess the industrialized degree of the entire procedure, which includes the design, prefabrication, construction, assembly, and operation of IB [34,35]. Furthermore, a systematic and objective assessment framework is lacking, which leaves a theoretical gap in the assessment of industrial building. The demand concerned with the sustainable development growth of the construction industry is also not well reflected.

Thus, it is essential to establish a reasonable assessment for the degree of industrialization in the construction industry. This study aims to fill this knowledge gap. The main objectives of this article are as follows:

(1) To establish the framework of IBA, which should include dimensions such as efficiency, economic factors, livability, safety, environmental factors, and social benefits;
(2) To assess the validity of the framework through data collected by assessing estimates of the framework and overall goodness of fit indices; and
(3) To test the positive impact among efficiency and the supplementary five dimensions (economic factors, livability, safety, environmental factors, and social benefits).

## 2. Literature Review

The scientific and rational IBA can enhance its implementation and growth. Setting up an effectual IBA is helpful for checking whether a building follows the necessities of construction development [7]. Considering the building assessment standards, existing green building assessments have been extensively recognized and used. In addition, environmental factors, together with the economic, social benefits, and safety factors included in green building standards [36,37,38,39], the IBA also considers the efficiency and livability of IB.

### 2.1. Efficiency

One of the benefits of IB is to upgrade efficiency. When prefabrication and on-site assembly is used subsequent to the design, the effectiveness of construction process can be enhanced, which lead to less construction time [40]. It will be necessary, however, to develop much higher requirements for design, construction, and management. Conventional design cannot fulfill the requirements of IB. Designers should have the ability to design structure, components, mechanical and electrical ornaments, prefabrication assemblage, and decoration [41]. Nevertheless, whether the prefabrication is done in the factory or via on-site assembly, more mechanical ornaments are used in prefabrication and constructive procedure, which is performed through assembly construction standards [42]. The conventional management mode is also not suitable for the industrialized construction mode [43], which needs informative management and communication integration for the entire procedure of design, prefabrication, assembly, construction, and operation [16,44].

### 2.2. Economic Factors

Economic factors have always been one of the key factors that impacts the growth of IB. In relation to traditional building, IB costs higher in some areas, for instance, preliminary inputs [45], more multifaceted designs, techniques costs [46], prefabrication costs [47], and additional transportation costs [48]. However, material consumption costs and operating and management costs of IB are comparatively lower [45]. The fact that cost of IB is greater compared with those of conventional building is verified by multiple cases and research, and it is caused primarily because of the higher cost of prefabricated components, transportation costs, and design consulting costs [47].

### 2.3. Structural Capacity

The structure of both conventional building and IB could be segregated into three types: steel structure [49,50], timber structure, and concrete structure [51,52,53]. The primary difference between conventional building and IB is the mode of construction instead of the structure [54]. IB is prefabricated in factories and assembled on site, while conventional building is constructed on site [55]. Steel structure is more appropriate for IB, whereas the most extensive application is concrete structure, particularly reinforced concrete (RC) structures [53,56]. The most ordinary components for prefabrication include prefabricated floor slabs [57], prefabricated façade, prefabricated beams, prefabricated columns, and prefabricated foundation [33].

### 2.4. Livability

The fundamental purpose of a building for individuals is living; thus, durability, safety, adaptability, and quality are taken into account. As compared with conventional building, IB can efficiently enhance the product accurateness, extend the service life of buildings, and enhance the durability of buildings [33]. The safety of IB is not remarkably distinct from that of conventional building, and it is usually believed that IB is equivalent to cast-in-situ in structural stabilities. The standardization of designs with fewer options are accepted in industrialized buildings, while customized user-oriented production is today’s trend, and more designers are more inclined towards building adaptability [58,59]. Owing to the stabilized quality of components manufactured scientifically, on-site assembly can remarkably minimize construction blunders and human error through minimizing defects in the quality [60].

### 2.5. Safety

Employment in the construction industry is generally unsafe, because working high above the ground in multifaceted environments [61] may lead to injury and sickness [62], earlier retirement [63], musculoskeletal grievances, and chronic infections [64]. Nevertheless, the construction of IB has modified the situation. Most of the work is not performed on site (because of factorial prefabrication [65], on-site construction has considerably declined [21]), mechanization is used for reducing risk and intensifying labor [66], and atmospheric conditions at the construction site are enhanced; therefore, the safety and health of employees are guaranteed [67] and the chances of accidents are declined [25].

### 2.6. Environmental Factors

IB can considerably improve anti-environmental pollution efforts. It can decrease the utilization of building materials by decreasing the generation of construction waste [68], harmful emissions, and environment pollution [69], which is also one of the aims for sustaining the growth of buildings [70,71]. Embodied energy (EE) is adopted for measuring overall energy utilized throughout the lifecycle of buildings. Foraboschi proposed that embodied energy relied primarily on the flooring system and that steel consumes more EE compared with reinforced concrete [72]. At the end of the lifetime of IB, it can be broken down into modules or components for the purpose of recycling and reusing [73].

### 2.7. Social Benefits

The social benefits of such buildings have gained the attention of researchers, primarily owing to the fact that the building process itself is a social activity [70]. The social benefits involve making all the participants satisfied with the design, construction, and operation of the project [74], enhancing the communication and innovation of technology, and improving the economic progress. For ensuring the demonstration effect and conducting a comprehensive assessment of the sustainability of the building, both Leadership in Energy and Environmental Design (LEED) in the United States of America (USA) and Building Research Establishment Environmental Assessment Method (BREEM) in the United Kingdom (UK) have established innovative standards for reflecting exceptional performance, which include procurement strategies, design features, management, and technological innovation [75].

## 3. Conceptual Framework and Theoretical Hypothesis

The clearest distinction between IB and conventional building is the transformation of the construction mode. Mechanical production enhances production efficacy that affects the economy of the overall construction and operational procedure, livability, safety and health of the employees, environment, innovation, and social benefits.

According to the concept and characteristics of IB, the framework of IBA consists of the following six dimensions: efficiency, economic factors, livability, safety, environmental factors, and social benefits, as shown in Table 1.

Based on the above study, the following hypotheses are presented:

**Hypothesis** **1.** *The efficiency of IB has a positive impact on economic factors*.

**Hypothesis** **2.** *The efficiency of IB has a positive impact on livability*.

**Hypothesis** **3.** *The efficiency of IB has a positive impact on safety*.

**Hypothesis** **4.** *The efficiency of IB has a positive impact on environmental factors*.

**Hypothesis** **5.** *The efficiency of IB has a positive impact on social benefits*.

## 4. Methodology

**Figure 1:**
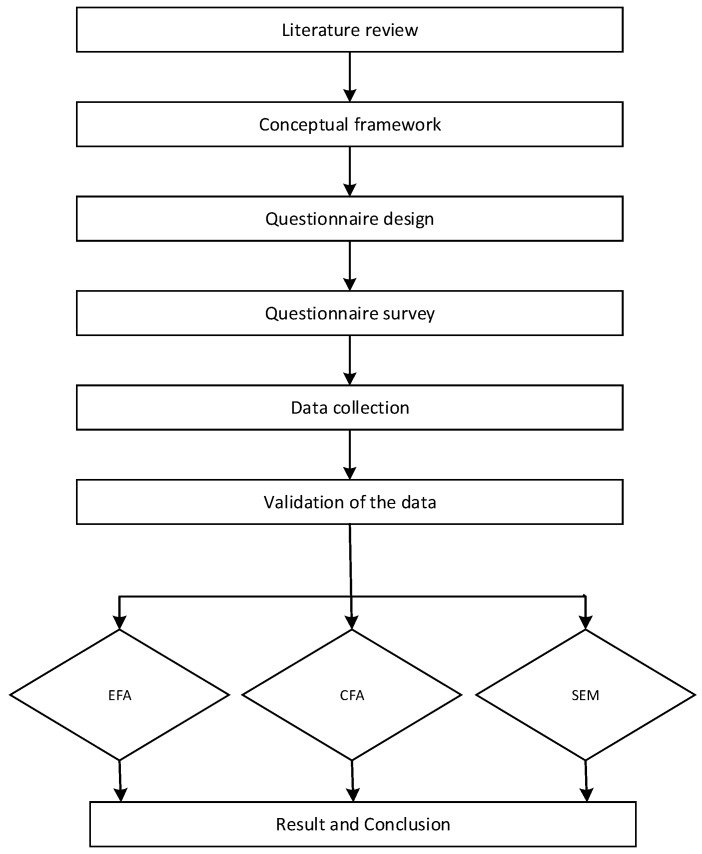
The research roadmap. EFA: exploratory factor analysis; CFA: confirmatory factor analysis; and SEM: structural equations model.

This paper studied the fundamental characteristic of IB and set up the conceptual framework of IBA according to the relevant theories and methodologies of building assessment. Then, research progress in questionnaire design, questionnaire survey, data collection, data validation, and data analysis are conducted, and the conclusion of this paper is drawn. The roadmap of this research is shown in Figure 1.

### 4.1. Literature Review

Based on the literature review, the definition, characteristics, and advantages of industrialized building were developed. This paper studies the basic aspects and classifications of building assessment. At present, there are few IBAs, and most assessments are concerned with safety and health [1,67], environmental impact [9,28], economical effect, and social benefit [26,30]. However, in addition to the above contents, rational IBA still requires the dimensions of efficiency and livability [33,42].

### 4.2. Conceptual Framework

Through the literature review, six dimensions of evaluation were established, which include efficiency, economic factors, livability, safety, environmental factors, and social benefits, resulting in a total of 23 evaluation indicators. We hypothesized that the efficiency of IB has a positive effect on the economic factors, livability, safety, environmental factors, and social benefits.

### 4.3. Questionnaire Design

We designed the questionnaire through conceptual framework. The questionnaire was composed of two parts. The first portion covered the basic information of all the participants, which includes age, type of work, employees, and work experience. The second portion related to the 23 indicators in the six dimensions of the IBA, as shown in Appendix A. The participants were requested to highlight values relating to the 23 indicators by means of a Likert five-item scaling method, in which 1 was very unimportant, 2 was less important, 3 was important, 4 was more important, and 5 was very important.

### 4.4. Questionnaire Survey

As IBA undertakes the entire procedure from design, prefabrication, assembly, construction, and operation, the questionnaire was distributed to developers, designers, contractors, engineers, component suppliers, and property managers. Because just 5% of projects in China are industrialized in construction at present, we have used snowball sampling in order to get data as much as possible. The initial 90 questionnaires were sent randomly to 15 designers, 15 contractors, 15 developers, 15 engineers, 15 component suppliers, and 15 property managers from China’s National Assembly Industrialized Base and the China Property Management Association from January to February in 2018. The questionnaire was performed through an online platform, and every participant was asked to send a web-link of the questionnaire to someone who is highly experienced in building industrialization.

### 4.5. Data Collection

A total of 772 questionnaires were distributed, and 295 valid questionnaires were received with an effective rate of 38.21%. The questionnaires were gathered from 31 provinces in mainland China (excluding for Hong Kong, Macao, and Taiwan), which includes 55 responses from designers, 52 responses from developers, 52 responses from engineers, 45 responses from contractors, 46 responses from component suppliers, and 45 responses from property managers, as shown in Table 2. The majority of the respondents have a minimum of five years of work experience in the construction industry.

## 5. Data Analysis

SPSS is a series of software products and interrelated services for statistical analysis, data mining, predictive analysis, and decision support tasks introduced by International Business Machines Corporation IBM [76]. Amos is used for analyzing of the structural equation model (SEM [77]), also known as the covariance structural analysis or the cause–effect model analysis. In this paper, SPSS 24.0 and Amos 24.0 were performed to process and analyze the data.

### 5.1. Reliability Analysis

Reliability analysis could be applied for measuring the consistency of investigative variables and scales in distinctive situations for measurement situations [78]. Moreover, in this study, the reliability coefficient of Cronbach’s Alpha is applied for examining the consistency of the variables in the questionnaire [79]. If we wish for the good reliability of the variable, the Cronbach’s Alpha coefficient should be greater than 0.7 [80]. The Cronbach’s Alpha coefficients of efficiency, economic factors, livability, safety, environmental factors, and social benefits are as follows: 0.876, 0.881, 0.893, 0.880, 0.922, and 0.913, respectively, where each value is greater than the standard of 0.7, showing that the variables have good internal consistent reliability, as shown in Table 3.

Table 4 illustrates the results of the frequency, means, standard deviations (SD), skewness, and kurtosis of the data. In this study, a survey of 23 items was adopted, and the responsive rate of every item varied from the value of 1 to 5. The results indicated that the mean values of the distinctive items were 3.06–3.68, and the standard deviation was 0.803–1.292.

### 5.2. Validity Analysis

Validity analysis is an essential part of empirical study. For questionnaires, content validity and structure validity are usually adopted for measuring [79]. Content validity refers to the appropriateness and rational consistency between the items and the tested variables. The questionnaire performed in this study is based on a literature review for showing the relation between the variables and the construction of correlation. Thus, this research puts the emphasis on structural validity. Furthermore, structural validation refers to the capability of items of measuring the variables. In this research, the data collected were tested by exploratory factor analysis (EFA) for proving the structural validity of the scale [78].

In general, EFA requires the feasibility test of factor analysis for satisfying both conditions. The Kaiser–Meyer–Olkin (KMO) measure >0.7, and Bartlett’s spherical test is significant (Sig. <0.005). SPSS24.0 was used for KMO and Bartlett’s spherical test, and the results are shown in Table 5.

The KMO measure is 0.883, which exceeded 0.7, and Bartlett’s spherical test was remarkable, the significance of which is 0.000. The findings indicated that the data were reliable with the requirement of EFA. Hence, further analysis was continued by employing principal component analysis (PCA) in extracting factor, and common factors were extracted under the situation of a characteristic root greater than 1. The varimax orthogonal rotation was applied to rotate factors in factor analysis. The PCA was performed through SPSS 24.0, and the result is shown in Table 6.

As is evident from Table 6, it can be determined that common factor 1 includes 5 items of VB1–VB5, common factor 2 includes 4 items of VE1–VE4, common factor 3 includes 4 items of VC1–VC4, common factor 4 includes 4 items of VA1–VA4, and common factor 5 includes 3 items of VF1–VF3, and common factor 6 includes 3 items of VD1–VD3, which is completely consistent with the previous conceptual framework. Common factor 1 is indicating economic factors, common factor 2 is indicating environmental factors, common factor 3 is indicating livability, common factor 4 is indicating efficiency, common factor 5 is indicating social benefit, and common factor 6 is indicating safety.

### 5.3. Confirmatory Factor Analysis (CFA)

**Figure 2:**
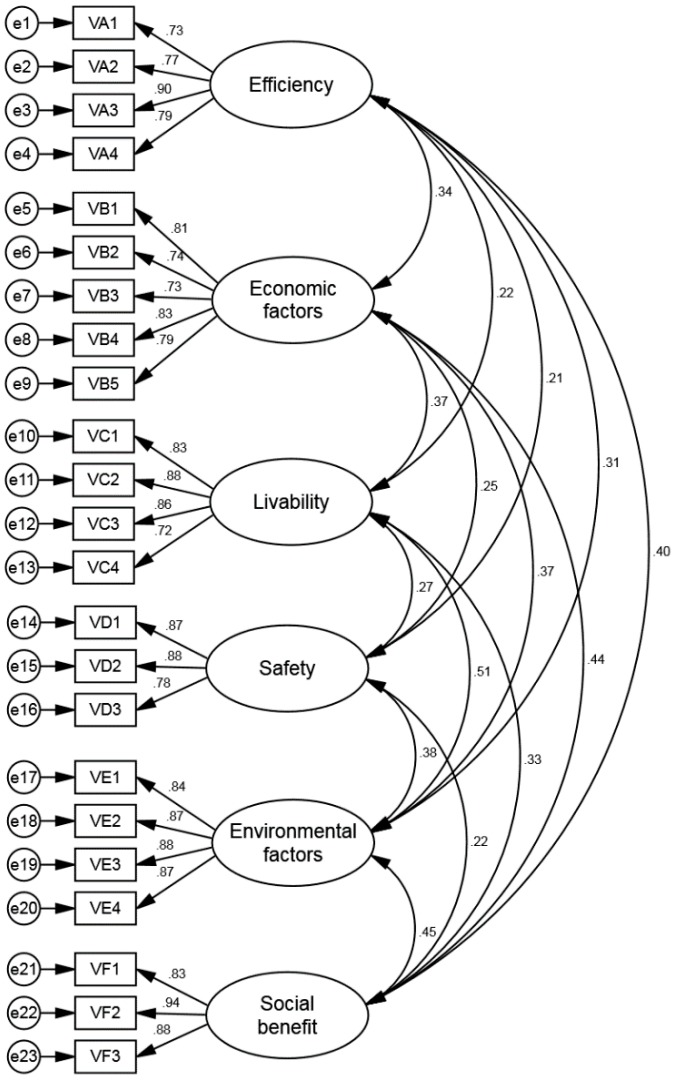
CFA model of IB evaluation.

Confirmatory factor analysis (CFA) is applied for testing the convergence validation of the internal items related to every variable; it aims at verifying the compatibility between the actual measurement of data and the theoretic framework [81]. The CFA model of IBA is illustrated in Figure 2. Testing the validity of CFA requires evaluating the model fit. This research has chosen some indices by which to assess the fitness of the entire model, including moderate contains chi-square (CMIN), normed chi-square (CMIN/DF), goodness-of-fit index (GFI), adjusted goodness-of-fit index (AGFI), root-mean-square error of approximation (RMSEA), incremental fit index (IFI), non-normed fit index (NNFI), and comparative fit index (CFI) [77].

Table 7 indicates that CMIN/DF is 1.326, which is less than 3. GFI, AGFI, IFI, NNFI, and CFI are greater than 0.9, and RMSEA is 0.033, less than 0.08. Each and every fit index imitates towards the ordinary standard of SEM. Thus, it takes into account that this model is a well-matched conceptual framework.

As it can be realized from Table 8, the standardized factor load of every item is greater than 0.7, and the remaining errors are positive and significant, indicating that there are no violated estimations. The component reliability (CR) of efficiency, economic factors, livability, safety, environmental factors, and social benefits were as follows: 0.877, 0.886, 0.895, 0.882, 0.922, and 0.916, respectively, where each value was greater than 0.7. The average variation extraction (AVE) was 0.642, 0.609, 0.682, 0.715, 0.748, and 0.785, where each value exceeds 0.5. These are compatible for the convergence validation standards. Model fit is also satisfactory, and every item is kept reserved for subsequent analysis.

### 5.4. Correlation Analysis and Discriminate Validity

The structure of all these dimensions and the conforming items was determined by means of validity analysis and reliability analysis, which is performed above. Subsequent to the calculation of the average score of all these dimensions, the correlation analysis was performed [82]. Correlation analysis is primarily for studying the correlation among variables, the range of which is from −1 to 1. The larger the absolute value, the closer the correlation among the variables. Discriminate validity refers to when distinctive methodologies are used for measuring different isomorphisms, the observed values should be distinguishable from each other.

A rigorous AVE method was adopted for evaluating the discriminate validity in this study. The diagonal of Table 9 is the root number of all these dimension’s AVEs, which should be higher as compared with the correlation coefficient for all pairs of variables [81]. Diagonal elements are higher as compared with off-diagonal elements in the corresponding rows and columns; thus this study has discriminant validity. The correlation coefficients among efficiency and economic factors, livability, safety, environmental factors, and social benefits are 0.343, 0.412, 0.292, 0.223, and 0.428, respectively; the *p* values are all significant. The findings indicated that that there is a statistically significant positive correlation among efficiency and economic factors, livability, safety, environmental factors, and social benefits.

### 5.5. Structural Equation Model (SEM)

**Figure 3:**
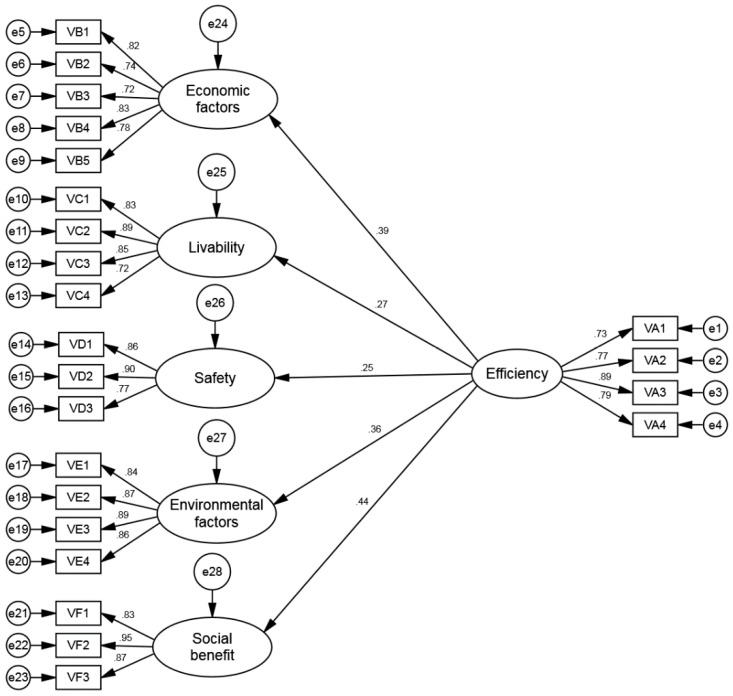
SEM of IBA.

Goodness-of-fit is required for the application of the SEM in order to validate the theoretic framework. The reliability extent of the expected overall variance estimate matrix with sample variance matrix expressed the closer association of the framework and sample. As the extent of the consistency is greater, there would be more closeness of the model with the sample [81]. For achieving this objective, scholars should consider the important statistical indicators of the SEM. The SEM framework of IBA is shown in Figure 3. In the evaluation of the model, we should take every indicator into account precisely when majority of the indicators fulfill the requirements; this will indicate the goodness-of-fit.

Table 10 indicates that CMIN/DF is 2.207, which is less than 3; GFI = 0.870, and AGFI = 0.840, where every value is greater than 0.8. IFI, NNFI, and CFI exceed 0.9, and RMSEA is 0.059, which is less than 0.08. Each and very index conforms to the standard of the SEM. Thus, it can be considered that this model is well fit.

As can be observed from Table 11, the standardization coefficient of efficiency towards the economic factors is 0.389, and the *p*-value is 0.001, which demonstrates that efficiency shares a statistically remarkable and positive correlation with the economic factors. The standardized coefficient of efficiency to livability is 0.272, and the *p*-value is 0.001, which shows that efficiency has a statistically significant positive correlation with livability. The standardized coefficient of efficiency to safety is 0.247, and the *p*-value is 0.001, which indicates that efficiency has a statistically significant positive correlation with safety. The standardization coefficient of efficiency to the environmental factors is 0.363, and the *p*-value is 0.001, which indicates that efficiency has a statistically significant positive correlation with environmental factors. The standardized coefficient of efficiency to social benefit is 0.437, and the *p*-value reaches a significant level of 0.001, indicating that efficiency has a statistically significant positive correlation with social benefit.

## 6. Discussion

Identifying the relationship among efficiency, economic factors, livability, safety, environmental factors, and social benefits is significant for IBA establishment. In this study, the relationship among efficiency and economic factors, livability, safety, environmental factors, and social benefits were explored. EFA together with CFA proved the accuracy of the conceptual framework. The SEM validated the positive impact of efficiency on economic factors, livability, safety, environmental factors, and social benefits. Particularly, the relationship of efficiency with social benefit was the strongest among all, as β = 0.437, *p* < 0.001, which showed that the efficiency of IB has a positive impact on social benefit (H5), and the false hypothesis that there is no relationship between efficiency and society is excluded. This indicated that the efficiency of IB has the most significant impact on social benefits. The enhancement of efficiency can promote the applications of new technologies and new management methods, give rise to spillover effects, and hence raises the satisfaction of the participants.

The second one is the relationship between efficiency and economic factors, β = 0.389, *p* < 0.001. Therefore, the hypothesis that the efficiency of IB has a positive effect on the economic factors (H1) is valid, and the null hypothesis is excluded. This shows that the efficiency of IB has a significant impact on the economic factors. The enhancement of efficiency can reduce the duration of construction, shorten labor input, save consumption of energy and building materials, and decrease the operating and maintenance cost.

The third is the relationship between efficiency and environmental factors, β = 0.363, *p* < 0.001. Therefore, the hypothesis 4 that the efficiency of IB has a positive impact on the environmental factors is valid, and the null hypothesis is again excluded. This indicates that the efficiency of IB has an obvious impact on environmental factors. The enhancement of efficiency can decrease wasteful emissions and save energy and resources. IB can contribute to recycling, which causing a reduction in environmental pollution and waste of resource.

The fourth is the relationship between efficiency and livability, β = 0.272, *p* < 0.001. So, the hypothesis 2 that the efficiency of IB has a positive effect on residential performance is valid, and the false hypothesis is excluded. This indicates that the efficiency of IB also has a certain impact on livability and the enhancement of efficiency can improve the quality of buildings, as well as the safety, durability, and adaptability of buildings.

Lastly, there is profound association between efficiency and safety, β = 0.247, *p* < 0.001. Therefore, the hypothesis 3 that the efficiency of IB has a positive impact on safety is valid, and the nullified hypothesis is excluded. This is showing that the efficiency of IB has a comparatively weak impact on the safety and health of employees. The changing mode of production and the enhancement of efficiency have altered the conventional working methods and operational conditions and can decrease the possibility of accidents occurring in construction to a certain extent and improve the safety and health of employees.

In previous research of building assessment, VE1 (waste reduction), VE2 (energy and resource savings), VE3 (recycling after the demolition of a building), and VE4 (environmental pollution reduction) were applied for evaluating the environmental performance of prefabricated school buildings [31]. VB1 (on-site construction cost), VB2 (operating and maintenance costs), VB3 (management cost), VB4 (prefabrication and transportation cost), and VB5 (consumption of building materials, energy, and resources) were applied to explore the basic cost composition of prefabrication and observe the effect of adopting prefabrication on the overall cost of real building projects [9]. VF1 (application of new technologies and management methods), VF2 (spillover effect), and VF3 (satisfaction of participants) were used for assessing the social performance of building [70,74,75]. This research is consistent with the framework of IBA in this paper. These assessments refer to several aspects in assessing the sustainability of IB, although none of them refers to the industrialized degree of IB. There is also no literature concerning the impact among the aspects.

The framework of IBA is not only evaluating the product, but also the process of IB. In fact, the positive impact of efficiency on all the above dimensions is in line with the practical use of IB. The industrialized construction process and the industrialized construction product are two different outlooks of a dichotomy [83]. Affected by the habits of the traditional Chinese construction industry, these housing developers put focus on producing profits by means of developing land and the management of finance throughout this procedure instead of actual construction mechanisms and the product itself [84]. Nevertheless, owing to the use of industrialized construction techniques and strategies such as prefabrication and standardization, the construction process is reduced and integrated, which gives rise to significantly reduced delivery time [54,85]. However, beneath the backdrop of China’s new urbanization, the integrated construction process enables a significant decline in the duration of project delivery, directly causing low financing costs and increasing economic benefits [86]. This benefit from the integrated construction process enables market organizations to invest more resources in the industrialized construction process as compared with the past period [87]. Furthermore, the higher degree of the industrialized construction process brings about the increasing value of the two dimensions of products and processes. Based on the perspective of the construction process, the application of advanced construction technologies and management strategies ensures the health and safety of the employees. Based on the perspective of the construction product, the enhancement of the construction logic gives rise to the perfect product performance [88]. However, efficiency and value correspond with the process and product of industrialized construction, which are two different outlooks of the dichotomy. It is the enhancement of the process-oriented efficacy that causes enhancement of the product-oriented value. The two perspectives of the dichotomy also offer the impetus to realize social benefits, together with innovation.

## 7. Conclusions

This research has constructed a conceptual framework of IBA for evaluating the industrialized degree of IB in China, and SEM was applied for exploring the impact on efficiency with all other five dimensions. The results of the questionnaire conformed to theoretic study and hypothesis. The results can be referred to as a solid reference point recognizing IBA in China. The key findings are as follows:

(1) The conceptual framework of IBA was constructed, which includes the following six dimensions: efficiency, economic factors, livability, safe, environmental factors, and social benefits. Additionally, it has 23 indicators in the above six dimensions.
(2) IB efficiency showed positive effect on the economic factors, livability, safety, environmental factors, and social benefits. Thus, efficiency is the main point of consideration in IBA.

The study is based on the current development stage of China’s IB. At present, China is in the initial stage of industrialized growth. With the enhancement of the industrialized degree, IBA and the relationship among the dimensions may be changed; therefore, it is necessary to track the investigation. This paper has established the framework of IBA without a detailed evaluation index and weight, and further research needs to be conducted.

## Figures and Tables

**Table 1:** A Conceptual framework for assessment of industrialized building (IB).

Dimension	Code	Indicators	References
Efficiency	VA1	Integrated design	[41]
	VA2	Integrated construction	[42]
	VA3	Integrated management	[16,43,44]
	VA4	Construction schedule	[1,9,18,21,40]
Economic factors	VB1	On-site construction cost	[1,47]
	VB2	Operating and maintenance costs	[9,45]
	VB3	Management cost	[9,45]
	VB4	Prefabrication and transportation cost	[1,9,47,48]
	VB5	Consumption of building materials, energy, and resources	[1,9,21,45]
Livability	VC1	Durability of building	[33]
	VC2	Safety of building	[33]
	VC3	Adaptability of building	[58,59]
	VC4	Quality level of the building	[1,18,21,60]
Safety	VD1	Safety of employees	[1,18,21,67]
	VD2	Health of employees	[1,62,64,67]
	VD3	Possibility of accidents in construction	[1,25,66]
Environmental factors	VE1	Waste reduction	[1,68]
	VE2	Energy and resource savings	[1,2,4,9,21,71]
	VE3	Recycling after the demolition of a building	[1,73]
	VE4	Environmental pollution reduction	[1,18,21,69]
Social benefits	VF1	Application of new technologies and management methods	[18,70]
	VF2	Spillover effects	[18,74]
	VF3	Satisfaction of participants	[18,75]

**Table 2:** Basic information about the samples.

Variables	Category	Frequency	Frequency (%)
Age	18–29	32	10.85%
	30–39	67	22.71%
	40–49	120	40.68%
	50–59	58	19.66%
	>60	18	6.10%
Type of work	Designers	55	18.64%
	Developers	52	17.63%
	Engineers	52	17.63%
	Contractors	45	15.25%
	Component suppliers	46	15.59%
	Property managers	45	15.25%
Number of employees	1–49	35	11.86%
	50–99	24	8.14%
	100–199	45	15.25%
	200–299	33	11.19%
	300–399	27	9.15%
	400–499	34	11.53%
	>500	97	32.88%
Working experience	1–5 years	19	6.44%
	6–10 years	28	9.49%
	10–15 years	30	10.17%
	16–20 years	59	20.00%
	21–25 years	90	30.51%
	26–30 years	42	14.24%
	>30 years	27	9.15%

**Table 3:** Reliability analysis in six dimensions.

Dimensions	Items	Cronbach’s α
Efficiency	4	0.876
Economic factors	5	0.881
Livability	4	0.893
Safety	3	0.880
Environmental factors	4	0.922
Social benefits	3	0.913

**Table 4:** Results of descriptive statistics.

Code	Frequency					Mean	SD	Skewness	Kurtosis
	1	2	3	4	5				
VA1	4	100	68	98	25	3.14	1.024	0.127	−1.120
VA2	20	44	104	102	25	3.23	1.027	−0.380	−0.298
VA3	20	49	95	99	32	3.25	1.071	−0.314	−0.489
VA4	15	32	122	96	30	3.32	0.972	−0.340	0.043
VB1	4	39	165	67	20	3.20	0.803	0.249	0.399
VB2	18	51	110	95	21	3.17	0.999	−0.284	−0.331
VB3	26	53	111	88	17	3.06	1.030	−0.285	−0.430
VB4	19	48	104	94	30	3.23	1.047	−0.276	−0.409
VB5	9	53	127	89	17	3.18	0.898	−0.127	−0.182
VC1	21	48	85	95	46	3.33	1.136	−0.321	−0.637
VC2	29	56	81	70	59	3.25	1.250	−0.169	−0.961
VC3	40	48	83	68	56	3.18	1.292	−0.180	−0.988
VC4	20	36	82	119	38	3.40	1.074	−0.560	−0.234
VD1	6	32	80	108	69	3.68	1.013	−0.443	−0.411
VD2	9	33	82	97	74	3.66	1.067	−0.451	−0.468
VD3	7	22	114	97	55	3.58	0.955	−0.253	−0.159
VE1	22	33	89	119	32	3.36	1.059	−0.586	−0.142
VE2	28	49	73	99	46	3.29	1.194	−0.362	−0.764
VE3	32	31	86	109	37	3.30	1.151	−0.535	−0.427
VE4	38	46	74	107	30	3.15	1.193	−0.395	−0.797
VF1	18	80	82	89	26	3.08	1.080	−0.039	−0.828
VF2	34	52	75	100	34	3.16	1.190	−0.318	−0.821
VF3	35	60	56	96	48	3.21	1.271	−0.271	−1.044

**Table 5:** Kaiser–Meyer–Olkin (KMO) measure and Bartlett’s test results.

Kaiser–Meyer–Olkin Measure of Sampling Adequacy		0.883
Bartlett’s spherical test	Approximate Chi-Square	4512.207
	df	253
	Sig.	0.000

**Table 6:** Rotated factor matrix of PCA.

Code	Factors						Communality
	1	2	3	4	5	6	
VA1	0.166	0.007	0.036	0.817	0.08	0.026	0.704
VA2	0.077	0.098	0.04	0.824	0.1	0.051	0.708
VA3	0.099	0.115	0.044	0.865	0.163	0.115	0.813
VA4	0.147	0.137	0.106	0.811	0.091	0.025	0.719
VB1	0.814	0.1	0.068	0.168	0.079	0.163	0.739
VB2	0.775	0.069	0.139	0.134	0.108	−0.001	0.655
VB3	0.742	0.114	0.156	0.058	0.145	0.135	0.63
VB4	0.844	0.109	0.086	0.093	0.122	−0.011	0.755
VB5	0.789	0.121	0.119	0.102	0.155	0.076	0.691
VC1	0.12	0.188	0.825	0.11	0.126	0.09	0.766
VC2	0.144	0.143	0.865	0.072	0.182	0.074	0.833
VC3	0.138	0.279	0.832	0.019	0.088	0.025	0.797
VC4	0.145	0.127	0.792	0.04	−0.065	0.15	0.693
VD1	0.076	0.22	0.104	0.049	−0.001	0.872	0.829
VD2	0.068	0.098	0.049	0.079	0.058	0.909	0.852
VD3	0.142	0.113	0.151	0.07	0.141	0.826	0.763
VE1	0.096	0.806	0.221	0.114	0.149	0.195	0.78
VE2	0.098	0.858	0.18	0.079	0.149	0.137	0.825
VE3	0.154	0.858	0.174	0.131	0.134	0.102	0.835
VE4	0.174	0.835	0.201	0.086	0.172	0.095	0.814
VF1	0.157	0.186	0.078	0.153	0.848	0.055	0.811
VF2	0.189	0.162	0.096	0.202	0.885	0.062	0.898
VF3	0.246	0.207	0.124	0.108	0.844	0.104	0.854
Eigenvalues	7.701	2.683	2.203	1.958	1.799	1.421	
Percentage of variance	33.483	11.665	9.578	8.513	7.82	6.179	
Cumulative percentage of variance	33.483	45.147	54.726	63.239	71.059	77.238	

**Table 7:** CFA model Fitness.

Fitting Index	Acceptable Range	Measured Value
CMIN		284.986
DF		215
CMIN/DF	<3	1.326
GFI	>0.8	0.925
AGFI	>0.8	0.903
RMSEA	<0.08	0.033
IFI	>0.9	0.984
NNFI	>0.9	0.981
CFI	>0.9	0.984

**Table 8:** Results of CFA.

Dimensions	Items	Non-Standardized Factor Load	Standard Error	CR (*t*-Value)	*p*	Standardized Factor Load	CR	AVE
Efficiency	VA1	1				0.733	0.877	0.642
	VA2	1.055	0.082	12.826	***	0.771		
	VA3	1.285	0.088	14.64	***	0.901		
	VA4	1.023	0.078	13.157	***	0.791		
Economic factors	VB1	1				0.812	0.886	0.609
	VB2	1.134	0.083	13.62	***	0.741		
	VB3	1.152	0.086	13.367	***	0.73		
	VB4	1.324	0.085	15.603	***	0.825		
	VB5	1.084	0.074	14.725	***	0.788		
Livability	VC1	1				0.833	0.895	0.682
	VC2	1.167	0.064	18.132	***	0.883		
	VC3	1.171	0.067	17.465	***	0.857		
	VC4	0.819	0.06	13.712	***	0.721		
Safety	VD1	1				0.874	0.882	0.715
	VD2	1.062	0.06	17.646	***	0.881		
	VD3	0.838	0.054	15.49	***	0.777		
Environmental factors	VE1	1				0.841	0.922	0.748
	VE2	1.165	0.062	18.747	***	0.87		
	VE3	1.139	0.059	19.139	***	0.881		
	VE4	1.16	0.062	18.634	***	0.867		
Social benefit	VF1	1			***	0.83	0.916	0.785
	VF2	1.249	0.062	20.236	***	0.941		
	VF3	1.253	0.066	18.938	***	0.884		

*** represents *p* < 0.001.

**Table 9:** Correlation analysis and discriminant validity.

Dimensions	Efficiency	Economic Factors	Livability	Safety	Environmental Factors	Social Benefit
Efficiency	0.801					
Economic factors	0.310 **	0.780				
Livability	0.192 **	0.341 **	0.825			
Safety	0.182 **	0.236 **	0.260 **	0.845		
Environmental factors	0.272 **	0.336 **	0.464 **	0.348 **	0.864	
Social benefits	0.343 **	0.412 **	0.292 **	0.223 **	0.428 **	0.886

** Correlation is significant at the 0.01 level (2-tailed).

**Table 10:** SEM fitness.

Fitness Index	Acceptable Range	Measured Value
CMIN		456.093
DF		225
CMIN/DF	<3	2.207
GFI	>0.8	0.870
AGFI	>0.8	0.840
RMSEA	<0.08	0.059
IFI	>0.9	0.948
NNFI	>0.9	0.941
CFI	>0.9	0.947

**Table 11:** Path coefficients of SEM.

Hypothesized Relationship			β Coefficient	S.E.	T	*p*	Supported or Rejected
Efficiency	→	Economy factors	0.389	0.059	5.824	***	Supported
Efficiency	→	Livability	0.272	0.082	4.164	***	Supported
Efficiency	→	Safety	0.247	0.077	3.768	***	Supported
Efficiency	→	Environmental factors	0.363	0.077	5.574	***	Supported
Efficiency	→	Social benefit	0.437	0.078	6.654	***	Supported

*** represents *p* < 0.001.

## Appendix A. Questionnaire

Part 1. Basic Information.

1. Your age is

□ 18–29	□ 30–39	□ 40–49	□ 50–59	□ > 60

2. Your work type is

□ Designers	□ Developers	□ Engineers	□ Contractors	□ Component suppliers	□ Property managers

3. The number of employees in the enterprise which you worked in is

□ 1–49	□ 50–99	□ 100–199	□ 200–299	□ 300–399	□ 400–499	□ >500

4. Your work experience in construction industry is

□ 1–5 years	□ 6–10 years	□ 10–15 years	□ 16–20 years	□ 21–25 years	□ 26–30 years	□ >30 years

Part 2. Please assess the following factors on a scale from 1 to 5, where 1 = “least important” and 5 = “most important”.

Efficiency					
VA1. Integrated design	1	2	3	4	5
VA2. Integrated construction	1	2	3	4	5
VA3. Integrated management	1	2	3	4	5
VA4. Construction schedule	1	2	3	4	5
Economic factors					
VB1. On-site construction cost	1	2	3	4	5
VB2. Operating and maintenance costs	1	2	3	4	5
VB3. Management cost	1	2	3	4	5
VB4. Prefabrication and transportation cost	1	2	3	4	5
VB5. Consumption of building materials, energy and resources	1	2	3	4	5
Livability					
VC1. Durability of building	1	2	3	4	5
VC2. Safety of building	1	2	3	4	5
VC3. Adaptability of building	1	2	3	4	5
VC4. Quality level of the building	1	2	3	4	5
Safety					
VD1. Safety of employees	1	2	3	4	5
VD2. Health of employees	1	2	3	4	5
VD3. Possibility of accidents in construction	1	2	3	4	5
Environmental factors					
VE1. Waste reduction	1	2	3	4	5
VE2. Energy and resource savings	1	2	3	4	5
VE3. Recycling after the demolition of a building	1	2	3	4	5
VE4. Environmental pollution reduction	1	2	3	4	5
Social benefits					
VF1. Application of new technologies and management methods	1	2	3	4	5
VF2. Spillover effect	1	2	3	4	5
VF3. Satisfaction of participants	1	2	3	4	5
